# The Role of Dimensionality in Understanding Granuloma Formation

**DOI:** 10.3390/computation6040058

**Published:** 2018-11-14

**Authors:** Simeone Marino, Caitlin Hult, Paul Wolberg, Jennifer J. Linderman, Denise E. Kirschner

**Affiliations:** 1 Department of Microbiology and Immunology, University of Michigan Medical School, Ann Arbor, MI 48109, USA; simeonem@umich.edu (S.M.); cshult@umich.edu (C.H.); pwolberg@umich.edu (P.W.); 2 Statistics Online Computational Resource (SOCR), Department of Health Behavior and Biological Sciences, University of Michigan, Ann Arbor, MI 48109, USA; 3 Department of Chemical Engineering, University of Michigan, Ann Arbor, MI 48109, USA; linderma@umich.edu; 4 Department of Computational Medicine and Bioinformatics, University of Michigan, Ann Arbor, MI 48109, USA

**Keywords:** 3D agent-based model, tuberculosis, granuloma, stochastic model calibration, 2D vs. 3D

## Abstract

Within the first 2–3 months of a *Mycobacterium tuberculosis* (Mtb) infection, 2–4 mm spherical structures called granulomas develop in the lungs of the infected hosts. These are the hallmark of tuberculosis (TB) infection in humans and non-human primates. A cascade of immunological events occurs in the first 3 months of granuloma formation that likely shapes the outcome of the infection. Understanding the main mechanisms driving granuloma development and function is key to generating treatments and vaccines. In vitro, in vivo, and in silico studies have been performed in the past decades to address the complexity of granuloma dynamics. This study builds on our previous 2D spatio-temporal hybrid computational model of granuloma formation in TB *(GranSim)* and presents for the first time a more realistic 3D implementation. We use uncertainty and sensitivity analysis techniques to calibrate the new 3D resolution to non-human primate (NHP) experimental data on bacterial levels per granuloma during the first 100 days post infection. Due to the large computational cost associated with running a 3D agent-based model, our major goal is to assess to what extent 2D and 3D simulations differ in predictions for TB granulomas and what can be learned in the context of 3D that is missed in 2D. Our findings suggest that in terms of major mechanisms driving bacterial burden, 2D and 3D models return very similar results. For example, Mtb growth rates and molecular regulation mechanisms are very important both in 2D and 3D, as are cellular movement and modulation of cell recruitment. The main difference we found was that the 3D model is less affected by crowding when cellular recruitment and movement of cells are increased. Overall, we conclude that the use of a 2D resolution in *GranSim* is warranted when large scale pilot runs are to be performed and if the goal is to determine major mechanisms driving infection outcome (e.g., bacterial load). To comprehensively compare the roles of model dimensionality, further tests and experimental data will be needed to expand our conclusions to molecular scale dynamics and multi-scale resolutions.

## 1. Introduction

Tuberculosis (TB) is an ancient disease that has re-emerged as the number one cause of death due to infection in the world [1]. Despite decades of research, the main factors that distinguish different disease outcomes—namely, clearance, clinical latency, and active disease—are not known. The hallmark of infection with the bacterium that causes TB, *Mycobacterium tuberculosis* (Mtb), is the development of lung granulomas that serve to immunologically restrain and physically contain the bacteria, despite often failing to fully clear it. Adding further complication is that etch human suffers the development of multiple granulomas, and each granuloma has been shown to have a unique trajectory [2], As granulomas serve as the site of infection dynamics, successful identification of those factors that contribute to whether a granuloma can or cannot control infection is key to eventual elimination of this disease worldwide.

**Figure 1.**
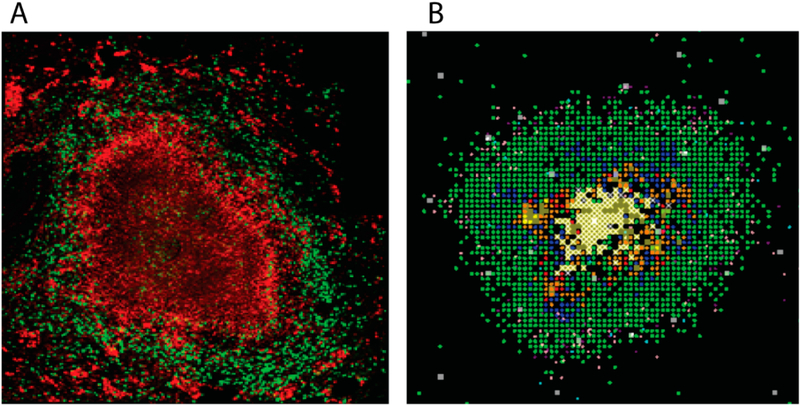
Non-human primate an. simulated granulomas. Panel (**A**) Non-human primate granuloma stained for macrophages (Red CD68) and T cells (Green CD3). The very center (black, no staining) is caseous necrosis (Image courtesy of Dr. Josh Mattila, University of Pittsburgh). Panel (**B**) *GranSim* outputs. 2D snapshot, zoomed in to show detail showing macrophages (green-resting, blue-activated, orange-infected, red-chronically infected), T cells (pink IFN-g producing, purple-cytotoxic, white-Tregs), extracellular bacteria (yellow), necrosis (brown areas).

There are several animal models that are used to study TB [3]. Mice were used as the animal of study for decades, but caseous granulomas do not develop in their lunge during infection, making them a poor choice for understanding granulomas and their role in human disease. Rabbit and guinea pigs do form excellent granulomas similar do those seen in humans, but there are limited reagents available to explore the immunological information encoded within. This leaves the study of non-human primates (NHPs) [4]. Figure 1A shows a typical NHP granuloma. These animals have a very similar disease course as that of humans, and their granulomas form in a similar fashion. In addition, the NHPs are outbred, so their individual diversity reflects that observed in humans.

Computational modelling has also been used over the past decade to explore the dynamic s of granuloma formation and function in TB (reviewed in [5]). Many of these models are developed as temporal models, tracking dynamics within lungs and/or lymph nodes (LNs) using ordinary or partial differential equations [6–10]. Later models track dynamics of granuloma formation and function in both space and time using agent-based models (ABMs) [11–14]. The main computational tool that has been used to study this is *GranSim*, which is an ABM that was developed in two-dimensions (2D) and is a hybrid formulation of an agent-based model and differential equations that tracks spatio-temporal granuloma development in real time (see Figure 1B and [15]]. Generated snapshot images can be strung together to create time-lapse movies. With regard to granulomas, the experimental data obtained from NHPs that can tie readily compared to simulated data are usually temporal in nature (cell counts, bacteria loads, etc.) or slices of tissue (essentially 2D) stained It}- immunohistochemistry (the experimental approach used to generate Figure 1A).

With both 2D granuloma experimental data as well as with simulations, the question remains as to how well they capture the 3D granuloma structures. While we are not poised to address the wet lab aspect of this question in this work, we can explore the relationship between our 2D simulations and a new 3D version of the model developed herein to predict how well, and under what circumstances, the 2D representation does a good job of predicting 3D behavior. Datasets derived from NHPs and humans on a whole-granuloma scale provide data from 3D in vivo experiments that we can use to calibrate the 3D model and validate our predictions.

We then are able to perform sensitivity and uncertainty analyses to predict what factors drive simulated granuloma dynamics in 3D and how they are similar to or different from those that drive dynamics in 2D. As questions pertaining to the importance and role of dimensionality are relevant to many other applications of 2D modelling found in the literature, for instance, that of tumors, this work can also prove helpful to a larger community.

### Previous Studies on 2D/3D

The questions regarding ABM modelling of lymph nodes (LNs) that we posed in our previous work [16] guide this work. Most models of LN structures have been created in 2D. Previously, we studied under what conditions a 3D version of a LN model provides different predictions than a 2D formulation.

In our previous 2D study of LNs [16], we defined a LN priming efficiency to be a measure of the capacity of the LN to expand the cognate T cell population during an immune response. It represents the ratio of the number of effector T cells leaving the LN to the number of naive cognate T cells entering the LN. Our 2D prediction for CD4+ T cells LN efficiency was ~5. Using our 3D ABM of a LN, we calculated that LN efficiencies for CD4+ T cells were as high as 15 [16]. We also assessed the similarities and differences between two additional measures: the match percentage and search time. Match percentage is the percent of cognate T cells that detect a matching antigen presenting cell before exiting a LN, and search time is the average time for a T cell to find the first matching antigen presenting cell in the LN. The 3D match percentage is ~90%, higher than the 40–60% that was measured in the 2D model [17], and the average 3D-search time is only 10 min, much less than our 2D model prediction of 2–5 h. Thus, in 3D it appears that more cells in the LN find a match, and do so more quickly, than would be predicted from the corresponding 2D model. This is largely due to the smaller distances between neighbors in the 3D model [17].

The measures examined above describe cells locating each other in space (either 2D or 3D), and thus it is not surprising that the sensitivity analysis identified different parameters between 2D and 3D that influenced model outcomes when those factors related to movement and dynamics of cell-cell interactions. As shown by the sensitivity analysis results in our LN studies, the parameters that most strongly affect our 3D model outputs [17,18] are different from those in the 2D model [17].

To our knowledge, since our LN study above there has been only one other study that compares results between a 2D and 3D model. In [19], Gadhamsetty et al. apply a cellular Potts model in different spatial configurations to investigate how the dimensionality of the space affects the functional response of cytotoxic T lymphocyte (CTL)-mediated killing. They found that saturation in a fully 3D environment is stronger than in a 2D environment, which is largely due to accompanying differences in the CTL-target encounter rates. As we determined [17,18], since the results are related to how cells find each other, it is not surprising that they also found that the major differences between outputs of 2D and 3D model formulations manifested for cell encounter rates.

The purpose of the work herein is to assess whether dynamics and mechanisms driving outcomes matter in 2D versus 3D for modelling TB granulomas and what can be learned in the context of 3D modelling that is missed in 2D. Additionally, using sensitivity analysis comparisons, we aim to determine which factors hold as significant across 2D and 3D, and which do not. This can assist in the choice between using 2D or 3D models in simulations going forward, as 3D models are much more expensive to run in terms of both simulation time and real time for research goals. Thus, having a way to parse which to use under what conditions will be extremely useful.

## 2. Methods

We integrated experimental data and computational model simulation to build and calibrate a 3D version of *GranSim*, our ABM of single granuloma formation during Mtb infection. The next sections briefly describe (i) granuloma immunobiology, (ii) 3D-ABM implementation of *GranSim*, (iii) uncertainty and sensitivity analysis and (iv) model calibration steps.

### 2.1. GranSim Description: Biology and Implementation

The immunobiology of Mtb infection has been described in many previous studies, both from our group and from others [5,20,21]. Here we first briefly review some key biological features that are captured in *GranSim*. Then we show how the biology is transformed into computational and mathematical constructs that recapitulate those features and allow us to postulate on the mechanisms driving granuloma formation and development.

#### 2.1.1. GranSim: Brief Immunology Background

Upon inhalation of Mtb, the host mounts an immune response which results in the localization of immune cells, namely lymphocytes, phagocytes (primarily macrophages) and polymorphonuclear cells (neutrophils and eosinophils), and fibroblasts in the lungs [22]. Each cell is recruited to the site of infection at different times, and the net result is the formation of a granuloma. Granulomas are emergent, complex, and dynamic structures. Granulomas allow the host to prevent Mtb from replicating and disseminating when they function properly. At the same time, Mtb has evolved to evade host killing mechanisms, and in some cases bacteria are able to persist within each granuloma for the lifetime of a host. The structure of Mtb granulomas in humans and NHPs is that of a cuff of lymphocytes surrounding an inner core of macrophages and bacteria and dead material called caseum (see Figure 1A).

Multiple granulomas can form within a single lung upon infection. However, it is noteworthy that granulomas evolve largely independently from one another, and *GranSim* represents a platform for studying the dynamics of individual granulomas in silico. The model can be used to generate insights into the emergence of such complex and consistent structures and how they function [5].

#### 2.1.2. GranSim: Implementation and High-Performance Computing

*GranSim* is a hybrid ABM that describes the formation and function of a granuloma during Mtb infection in the lung. *GranSim* captures the cellular behavior of macrophages (resting, activated, infected, and chronically infected), T lymphocytes (CD4+ labeled as Tgamma, CD8+ labeled as Tc, regulatory labeled as Treg), CD8+, and regulatory), and bacteria (intracellular replicating, extracellular replicating, and extracellular non-replicating). It also incorporates molecule dynamics, principally degradation and diffusion of the relevant cytokines Tumor Necrosis Factor (TNF), Interferon gamma (IFN-γ) and interleukin 10 (IL-10) and the chemokines (i.e., CCL2, CCL5, CXCL9); these are modelled using partial differential equations. Bacteria can be simulated either as agents or as continuous entities.

Although granulomas are 3D structures in the lung, due to the high computational costs associated with simulating in 3D, *GranSim* has been developed and curated since 2004 in 2D [11,13,23–28]. Typically, the environment is a grid of 100 × 100 microcompartments, each with size 20 × 20 μm, for a total size of 2 mm^2^. The size of each micro-compartment has been determined by matching the grid compartment to the average size of the largest immune cell, macrophages, reconciling their average speed with the iteration clock of *GranSim* (10 min) [11]. Each micro-compartment can accommodate at most one macrophage and one T cell (typically half the size of a macrophage), or two T cells. Simulations follow 2000–3000 cells and 5 diffusing molecules as a granuloma forms over a period of 200 days following infection. Diffusion is typically the most time-consuming process of the computation [29], even when using a fast Fourier Transform (FFT)-based algorithm implemented using the FFTW library [30]. The diffusion time step is 60 s.

**Figure 2.**
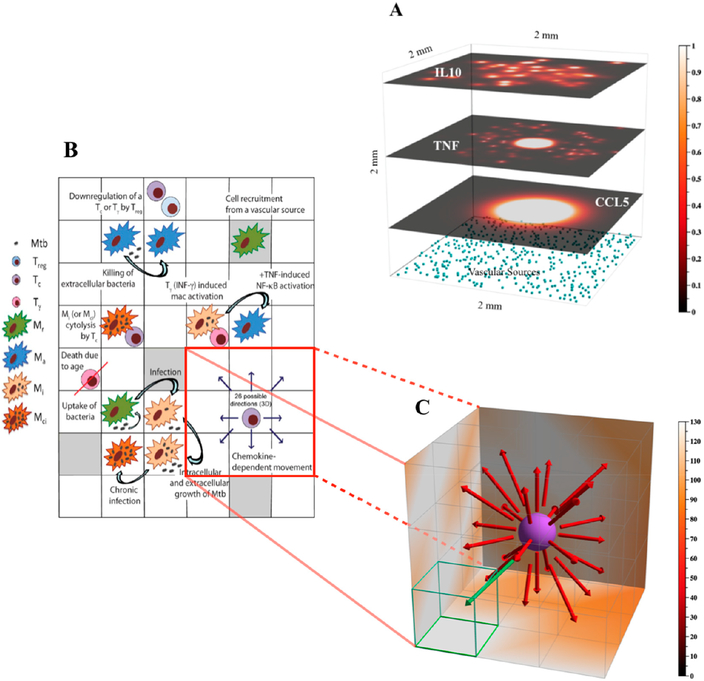
Environment and rules for the 3D version of *GranSim*. Panel (**A**) Example of molecular diffusion of different molecules in the *GranSim* environment. The top three layers show three different molecules (top layer—IL-10, middle layer—TNF, lower layer—CCL5) diffusing in a 3D environment of 2 mm^3^ with 100 × 100 × 100 microcompartments. The relative concentrations of the molecules in each microcompartment are shown as a heatmap. The three layers correspond to 2D mesh xy-slices at day exp1392 (from our 1500 runs, as described in Section 2.3 Model Calibration and shown in Figure 3), for z-values of 94, 59), anal 24, respertively. The xyz-locations of vascular sources (light blue cubes) are also shown, restricted here to those with a z-value of 0. Vascular sources denote sites at which new cells can enter the grid. Shown axes are xyz-positive. Panel (**B**) Schematic describing key agent rules captured in the 3D *GranSim* model. Panel (**C**) Zoomed-in 3D visualization of a subsection of the 2D schematic shown in (**B**), in which cell movement rules are emphasized. Movement of a simulated cell type (in this case, a cytotoxic T cell, Tc) is dependent on chemokine concentrations, and a cell will either stay in its current compartment or move to one of tire neighboring 26 compartments (i.e., its Moore neighborhood). In this example, the green arrow indicates the direction of highest chemokine concentration and thus the most probable direction of cell movement (i.e., to the compartment outlined in green).

This study represents the first attempt to simulate a 3D TB granuloma in the lung (Figure 2). The initial environment now comprises a grid of 100 × 100 × 100 micro-compartments which represents a physical size of 2 mm^3^ (see Figure 2B for details). The grid is Initialized with physiologically appropriate densities of vascular sources (Figure 2A) and resident macrophages (see Table S1 in the Supplementary Material for details of the parameters *sourceDensity* and *intiDensity*). More details on *GranSim* rules and specifications can be found in Figure 2, on our webtite [31].

Our implementation of the 3D version of *GranSim* is integrated with the 2D version in a single source code (see [31]). This upgraded implementation uses C++ features to generalize sections of the code that would otherwise be specific to the dimensions. For example, for iterating over the simulation grid, or local neighborhoods within the grid, the 2D version used 2 nested loops and the 3D version used 3 nested loops (see pseudocode in Box 1).

In order for our software to be cross-platform among Linux, Windows, and MacOS X, we used the Qt application framework for individual runs [32]. The Qt platform facilitates the development of a graphical user interface (GUI) that has flexible display options, including a graphics window displaying running simulation state, with zoom, pan, control of the graphics display, and the ability to display simulation summary statistics or detailed information about specific grid locations. The various visualization techniques including color mapping, isolines, height plots, and agent visualization have been implemented using OpenGL [33].

#### 2.1.3. DataTank for 3D Visualization

We used DataTank, an object-oriented programming environment providing large dataset support and visualization tools, to generate many of the figures herein (see [34] for details). Created and continually updated by D. Adalsteinsson (University of North Carolina at Chapel Hill), DataTank is a powerful visual and numerical work environment. It offers 80 variable types to handle data, approximately 2000 computational actions or “modules”, a wide variety of input and output formats, and 7 different drawing environments, as well as the possibility of launching one’s own programs from within DataTank. The advantages of this numerical work environment are particularly evident in 3D models, with regard to both speed and visualization possibilities.

DataTank enables us to visualize the 3D spread and density of cytokine/necrosis gradients in space and time, to view internal “slices” or subsections of a granuloma, and to conceptualize the evolution of multiple types of cytokine distributions simultaneously. To do so, we first ported temporal 3D datasets generated using *GranSim* into DataTank: cell xyz-position data, cytokine/chemokine grids, and the grid corresponding to the granuloma necrotic area. Using DataTank modules, we then created and automated a pipeline through which these imported data files can be read, manipulated, analyzed, and visualized over time. We wrote a linked, external C++ program to select and distinguish among cells by type and state as part of this pipeline. The implementation of semi-transparency, slicing, cropped display of variables within figures, rotation features, and thresholding were of particular relevance in this work, as described in appropriate figure legends. Recreating visualizations such as those in Figures 4 and 5 using DataTank requires some familiarity with the software, which users can obtain through the sample files, tutorials, and help manual provided with the software, as well as through working with the detailed and friendly user interface. Although we import data output in the form of CSV files from *GranSim* into DataTank, many different types of data files are compatible with DataTank.

### 2.2. Uncertainty and Sensitivity Analysis

We used statistical techniques for assessing model uncertainty and to apportion the impact of parameter variability towards outcomes of interest, such as bacterial burden. Uncertainty analysis was performed by sampling the parameter space with a Latin hypercube sampling (LHS) scheme. Sensitivity analysis was performed by a generalized correlation coefficient, namely the partial rank correlation coefficient (PRCC). The details on LHS and PRCC procedures are available in our review [35] and website [36]). Our scripts for using MATLAB are also available on the website. We also use LHS to calibrate the 3D ABM (described in [9] and in the next section).

### 2.3. Model Calibration

To calibrate our 3D model, we used published experimental data on bacterial loads per granuloma (also known as Colony Forming Unit-CFU) in NHPs [28,37]. At a future stage in model development, when experimental data are available on immune cell composition (macrophages and T cells) as a function of time, these cell types can also be included in the calibration criteria. We will use CFU in the rest of the manuscript to refer to bacterial levels per granuloma. To have an assessment of 3D model uncertainty, we conducted a large study varying 90 parameters in medium to large ranges (see Table S1 in the Supplementary Material for details). This uncertainty pilot study comprised 1500 parameter samples, each one simulated 5 times to control for stochastic uncertainty, for a total of 7500 simulations up to 100 days post infection. We then followed two main criteria for model calibration: (i) Stage 1 (temporal) CFU match, and (ii) Stage 2 (spatial) proper granuloma formation (c.f. Figure 1A). The second stage was implemented by examining the time-lapse simulations for proper structures as we have done previously for 2D [11,13,23–28].

The model calibration protocol used here aims to limit the stochastic variability embedded in the ABM implementation. Stochastic variability means that solving the ABM with the same parameter set (i.e., controlling for structural/epistemic variability induced by changing parameter values) will not return the same outcome as in deterministic systems (see [35] for details, definitions, and examples). Upon limiting stochastic variability, we also fulfill at the same time (i) classical model “fitting” criteria (i.e., matching data over time) and (ii) matching granuloma spatial cell organization.

#### 2.3.1. Stage 1—Temporal Criteria and Controlled Stochastic Variability

For the temporal criteria, we selected in silico trajectories that fell within the minima and maxima CFU from NHP CFU experimental data for the first 100 days post infection. Since the in silico trajectories consist of 5 replications using the same parameter values, the added constraint to pass Stage 1 was that all the 5 replications fell within the range of the experimental data. If any of the 5 replications failed, the sample was discarded.

#### 2.3.2. Stage 2—Spatial Criteria

For the spatial criteria, we generated snapshots at different time points for each of the in silico trajectories that passed Stage 1. Then, we selected in silico trajectories that resulted in properly formed granulomas. A properly formed granuloma is loosely defined as a collection of cells and bacteria with a clearly delimited structure and boundaries. Snapshots that did not clearly suggest such boundaries were discarded. While the generation of in silico granuloma snapshots is automated, the selection process is mostly manual at this point (see Discussion for details).

We ran this large experiment partly on our local server and partly using national resources (see Acknowledgments) for a total of ~0.5 M CPU h, with a computational time for a 3D run of 40–200 h, depending on the number of agents on the grid (see Table 1 for details). We then used the same set of 1500 parameter samples generated for the 3D platform to perform 2D in silico simulations. Since the 2D runs are not computationally costly (as shown in Table 1), we performed 10 replications for each parameter sample, for a total of 15,000 in silico granulomas. These estimates are for an in silico infection of 100 days post infection. We used both in silico repositories for model calibration and sensitivity analysis, with the main goal of comparing 2D and 3D resolution results.

## 3. Results

The next sections present model calibration and uncertainty and sensitivity analysis results. We will also highlight many 3D visualization tools and the insights they generate into granuloma formation and development.

### 3.1. 2D Scaling Factor Accurately Predict CFU per Granuloma Measures in 3D

**Figure 3.**
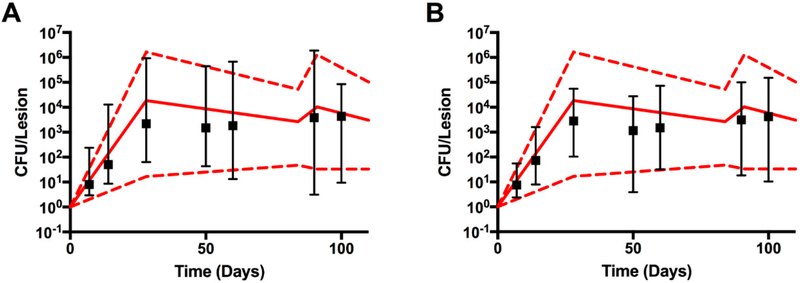
CFU data from 3D and 2D model simulations. The 22 simulations which met our two criteria: (i) temporal matching and controlled stochastic variability, and (ii) proper granuloma structure, are included. The red dotted and solid lines represent the min-max and median NHP CFU data, respectively. The black box plots (median, b lack square and 95% confidence intervals) represent *GranSim* CFU values. Panel (**A**) 3D results. Panel (**B**) 2D results. Data in Panel (**B**) were scaled from 2D to 3D using a scaling technique from [38].

As described in Methods, CFU calibration was done in 2 steps. First, we matched the CFU per granuloma from the experimental data on NHP subjects. It is important to note here that since there are no longitudinal experimental data available on bacterial burden per granuloma, the model “fitting” is artificial and combines data collected on each NHP at the time they have been sacrificed over 100 days post infection. The second step checks if proper granulomas are formed within the temporal dynamics of each in silico granuloma that passed step 1. Twenty-two (out of 1500) in silico granulomas passed both steps (Figure S1 in the Supplementary Materials shows some of the granuloma snapshots of these 22 runs). Figure 3A shows the temporal courses of these 22 granulomas. We also show (Figure 3B) the equivalent set of granulomas but in the 2D resolution of *GranSim*. For consistency, the same parameter files were used to generate 2D and 3D CFU time courses. The results are similar, indicating that with a scaling factor to scale the 2D model output to 3D data (see [38] for details on the scaling factor used here) a 2D representation can capture the 3D dynamics, at least if we consider median trajectories for bacteria, which are concentrated mostly at the center of the granulomas. Figure 3 also suggests that if the stochastic variability is controlled under the 3D resolution, it will be preserved for 2D as well.

### 3.2. Sensitivity Analysis Results: 2D and 3D Both Predict the Same Main Mechanisms Driving CFU Progression

We performed sensitivity analysis on both the 2D and the 3D in silico granulomas. The outcome of interest was the bacterial burden (CFU) per granuloma at time points: days 7, 14, 28, 50, 60, 90, and 100 post infection (the time points are based on the availability of the NHP experimental data). Table 2 shows all of the PRCC results.

Both the 2D and 3D PRCC results consistently predict the same most important parameters, and their magnitudes and signs are consistent across the two resolutions. It is important to note that this consistency of PRCC results is mostly true for positive PRCCs, which are also the largest ones. For example, the intracellular Mtb growth rate (e.g., *growthRatelntMtb)* is consistently selected at the most important mechanism driving; CFU in single granulomas in both 2D and 3D, with values ~0.9.

#### 3.2.1. Importance of Dimensionality in Recruitment and Movement

Parameters that directly affect cell recruitment, such as the vascular sources of the lung grid *(sourceDensity)* or the maximum probability of recruiting either a macrophage *(maxRecPro.)* or a Tgamma cell *(Tgam_maxRecProb)*: are consistently important in the 2D setting (negative correlation), suggesting that a potential crowding effect, caused by the emergence of many cells in the granuloma, might affect recruitment much more in 2D than in SD. This finding; is confirmed by this PRCC (negative correlation) for the parameter *probMoveToMac* (the probability of a T cell moving into a compartment already containing a macrophage), as it is consistently significant in 2D but absent in 3D. A crowding effect is likely stronger in 2D due to the lower degrees of freedom for agent movement in its Moore Neighborhood (8 vs. 26 possible directions or not moving). Enhancing recruitment as well as facilitating movement of T cells in 2D seems an effective mechanism to reduce bacterial levels. In contrast, the 3D setting does not gain any significant advantage when these two mechanisms are increased (e.g., more vascular sources, higher rates of recruitment for macrophages and T gamma cells).

A regulatory mechanism triggered by cell recruitment that appears to be consistently at play in both 2D and 3D after 4 weeks post infection is the (max) recruitment of regulatory T cells: the more Tregs are recruited, the higher the CFU per granuloma. This effect is more pronounced in 3D.

#### 3.2.2. Enhanced TNF Concentration on the Grid Correlates with Better Outcome Independently of the Dimensionality

Several TNF-related parameters show a significant PRCC value, both in 2D and 3D, suggesting an important role associated with TNF regulation. TNF is a molecule that does 4 key things in TB immunobiology, and thus in our models: (1) TNF acts to enhance recruitment of immune cells during infections, (2) TNF activates macrophages that make chemokines, (3) TNF allows for bacterial killing by macrophages, upon activation, and (4) TNF itself can induce death (apoptosis) of infected cells also killing intracellular bacteria. Overall, we find that every mechanism/parameter that is aimed at increasing TNF levels on the grid is associated with lower bacterial levels. Some mechanisms are important early during infection (within the first 4–8 weeks), such as the rate of TNF-induced apoptosis *(kApoptosis)* and the TNF threshold for TNF-induced apoptosis *(thresholdApoptosisTNF)*, as well as the scaling factor, boundTNFR1 *(estIntPartitionTNF)*, for coarse grained internalization. The TNF degradation rate constant (*kDeg*) and a scaling factor that accounts for the coarse-grained TNF dynamics *(estConsRateTNF)* only significantly affect bacterial levels after ~50–60 days post infection. The rate of NFkB activation *(kNFkB)* also significantly affects bacterial numbers after 50–60 days post infection, but only in 3D. Although each of these TNF-related mechanisms show significant PRCC values, their magnitudes are not as strong as the PRCC associated with the intracellular bacteria growth rate that dominates all of the PRCC sensitivities (e.g., ~0.9). Taken together, these results suggest a combined effect that points to the need for a better representation of the TNF molecular scale dynamics in 3D, as we discussed in detail in several of our previous 2D studies [12,24,39].

#### 3.2.3. Visualization Tools and Techniques Enhance Understanding of the 3D Granuloma and Lung Environment

**Figure 4.**
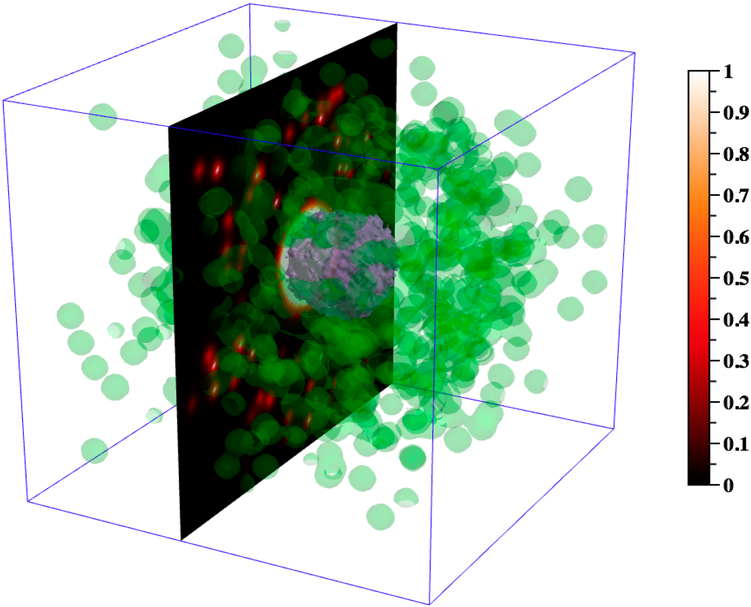
Visualizing the simulated TNF chemical gradient at day 50 post infection for one simulation, using DataTank. This figure; depicts the TNI gradient at day 50 using; two different methods: (i) a 2D, yz-slice at x = 50, and (ii) 3D level sets. In (i) we show the; 2D TNF gradient using the color bar on the right to define concentration intensity. In (ii) we use 3D level sets to depict the 3D spatial distributions of two biologically relevant TNF concentrations for macrophage recruitment. The green 3D surface encloses grid compartments in which the TNF concentration is 0.05 or greater (i.e., level set = 0.05). The 0.05 value represents the minimum concentration that macrophages can sense, and thus a concentration below this value is not detectable by macrophages. The purple 3D surface encloses grid compartments in which the TNF concentration is 50 or greater (i.e., level set = 50). The 50 value represents the maximum concentration that macrophages can sense, i.e., the concentration at which a compartment is considered “saturated” for macrophage sensitivity (~99% saturation according to Michaelis-Menten kinetics).

**Figure 5.**
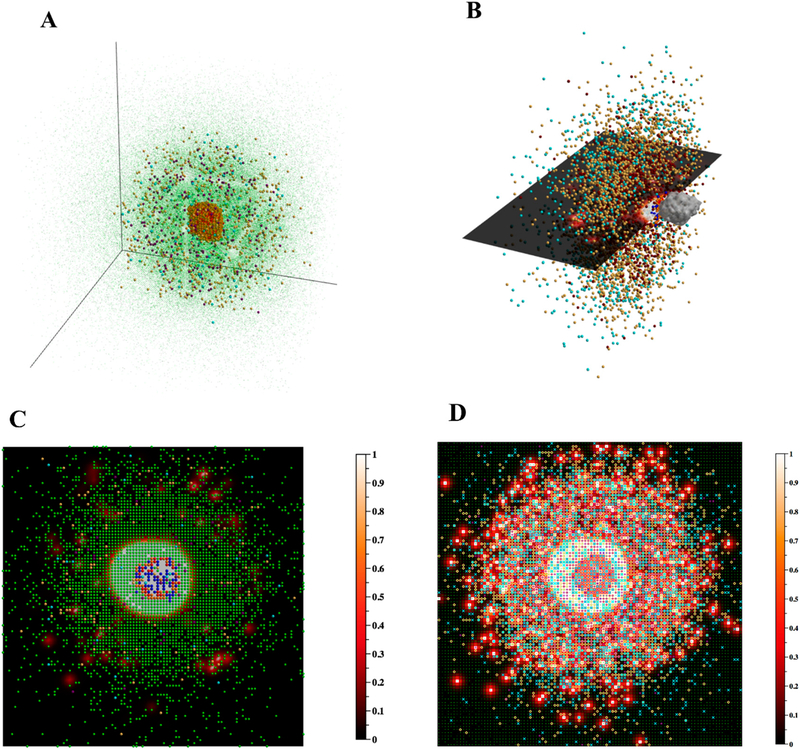
Visualizing the agents in 3D. (**A**) The 3D xyz-locations of the agents are shown at day 29 post infection. Macrophages (green—resting, orange—infected, red—chronically infected, blue—activated), T cells (yellow-orange—Tgam, maroon—Tcyt, cyan—Treg), extracellular Mtb (not shown), necrosis/caseum (not shown). For better visualization, resting macrophages are plotted at a smaller size than other cell types so as not to obscure; other cells. Shown axes are xyz-positive. (**B**) To Vetter visualize the interior of the granuloma, displayed agents from (**A**) are restricted to the sub-region [0,99] × [0,49)] × [0,99] of the xyz-grid and resting macrophages are not shown. A 2D mesh xy-slice (also restricted to this sub-region) displays the current TNF gradient at z = 50. Grid compartments in which there has been at least one cell death contributing to caseum formation are enclosed by a light gray 3D surface. The display of this caseum ‘surface’ is not restricted to the above sub-region, and instead reflects the entire 100 × 100 × 100 grid. Data correspond to day 32 post infection. (**C**) The 2D xy-slice from (**B**), rotated as though viewed from above, and no longer restricted to the above sub-region, instead drawing from the entire 100 × 100 xy-grid. All agents with a z-value of 50 are shown, including resting macrophages. (**D**) In contrast to (**C**), in which we show a single z-slice of the 3D data, here we show an alternative way of visualizing 3D data in 2D space, a projection of the agents and TNF gradient onto the 2D xy-plane z = 0. Data correspond to day 32 post infection.

Effective visualization of a biological system, whether through experimental or computational methods, is often crucial to further our understanding. Particularly in the case of biological systems at small scales, mathematical modeling and visualization of simulated data can lend insight when intuition or experimental methods are limited. Visualizing model output may expose non- or counter-intuitive spatial configurations, temporal processes, or agent behaviors. In Figures 4 and 5, as well as in Figure S1 in the Supplementary Materials, we present useful visualizations for the 3D granuloma and lung environment that increase our understanding of this system.

A 3D computational model affords us the ability to visualize concentration gradients in 3D both spatially and temporally. Whereas experimental datasets are more limited, *GranSim* generates high-resolution cytokine gradient data and returns the molecule concentrations in each grid compartment as cytokines diffuse over time. Through visualizing and analyzing this data, we can gain better intuition about how cytokines diffuse within the grid—for example, where (spatially) and when (temporally) over the course of the simulation the highest concentrations are present. Thus, we are better equipped to generate informed hypotheses regarding phenomena such as the relationship between cytokines and agent movement. Figure 4 exemplifies two of the visualization methods for concentration gradients possible in DataTank. Through the use of level sets, we can view the TNF concentration gradient at day 50 as 3D surfaces, something that is not possible through experimental techniques. As described in the legend, by thresholding these two level sets to biologically meaningful values, we can gain a qualitative, visual sense for the 3D space in which TNF concentration gradients play an active role in macrophage recruitment. Thus, in our model, it follows that macrophages are not able to sense preferential TNF concentration gradients within the purple surfaces or outside of the green surfaces. An animated version of this figure, in which the 2D yz-slice of the TNF gradient shown in Figure 4 moves through x-space, can be viewed in [31].

In Figure 5, we present ways to visualize the 3D agent data using DataTank: the entire granuloma, half of the granuloma, a single slice of the granuloma, and a 2D projection of a 3D granuloma. Each form of visualization informs, augments, and complements the others, the goal being to use multiple types of visualization to learn about agent spatial orientation of cells wiihin a granuloma. Figure 5A shows; the overall 310 structure of the simulated granuloma, Figure 5B provides a sense of the overall 3D structure while also showing part of the interior of the granuloma, and Figure 5C,D present two different ways of viewing the data in 2D space. Viewing 3D data through the lens of a 2D projection as in Figure 5D offers a useful alternative form of visualization to Figure 5C, in that it better captures the xy-spatial range of the; agents and global concentration of the TNF gradient. However, it also loses local information apparent at the single slice scale; for instance, the degree of agent crowding, or the lack of uniformity in agent or cytokine spatial distribution. It is worth noting that since the 2D model operates within a grid in which agent movement along the z-axis is restricted, neither Figure 5C nor Figure 5D are equivalent to images generated with data from the 2D version of *GranSim* (Figure 1B). However, Figure 5C offers o useful correlation with experimental images such as IHC (Figure 1A), and together with Figure 5D suggests that the method used in visualizing and analyzing 3D biological systems in 2D space matters, and furthermore, that multiple types of visualization can afford a more complete picture.

## 4. Discussion

Most human physiological structures are in 3D. However, many mathematical and computational models are built to describe events in 2D. From both a mathematical analysis and computational efficiency point of view, such an approach makes sense; however, when making predictions about a 3D system, it also necessitates an acknowledgement of the degree to which the 2D representation reflects and captures the 3D biological system. This is also true in experimental studies in which cross-sections (such as the immunohistochemistry tissue slice shown in Figure 1A) are often examined due to ease of protocols and tools. Clearly, one can glean important spatial information from that image; however, the role that the spatial context of cells within the entire structure plays likely cannot be discerned from this single slice.

In this work, we addressed the question of whether using a 3D version of a granuloma model is best practice, or if using a 2D representation is acceptable and, if so, under what conditions. This study, as well as previous work by our group and others, suggests that it is possible to use a 2D representation in some instances [17–19]. Our experience suggests that what is acceptable to coarse grain and simulate in a 2D ABM versus using a 3D ABM is based largely on space-limited crowding effects in 2D as compared to 3D, as well as for mechanisms where cells have to locate each other in space and time. This conclusion is based on our sensitivity analysis, which found that the sensitivity of the model to parameters that determine behavior in our 2D versus 3D ABM was most different for parameters that govern cell recruitment and cell movement. These processes happen when healthy host cells need to locate each other to communicate or healthy cells must locate infected cells or foreign invaders to kill those cells or activate them to do killing of their internal pathogens. Within a granuloma, these processes certainly do occur, but as cytokines and chemokines are playing the major role of cell signaling and directing, these signals are more strongly correlated with cell behavior dynamics. Also, whether to use 2D versus 3D may in large part depend on how the questions one is asking are focused; i.e., Whether they are focused on cell-cell interactions or on other system behaviors.

An ABM is a suitable model to capture this type of behavior; however, we argue here that determining the dynamics is key to knowing what dimension is appropriate and when. This does not negate the fact that there are also important trade-offs to consider when choosing between a 2D and 3D modeling approach, such as level of detail, number of runs required to perform analyses, computational run time, and the time and cost of access to high performance computing resources.

Our study represents the first attempt to (i) calibrate a 3D ABM describing TB granuloma model and (ii) assess if a 2D ABM representation that uses the same parameter values will reach similar results, at least in terms of matching available experimental datasets and returning similar sensitivity analysis results.

*GranSim* is an ABM, and thus by construction is a discrete, stochastic formulation. Figure 3 suggests that if the stochastic variability is controlled under the 2D resolution, it will be preserved for 3D as well. Further tests will be performed to validate this working hypothesis, since we calibrated the model based only on experimental datasets available to us, namely CFU per granuloma. Again, the bottleneck is computational costs associated with performing such tests. In terms of sensitivity analysis, our results are in agreement between the 2D and 3D formulations of the ABM for the main mechanisms driving CFU, suggesting that a 2D resolution is adequate to represent the 3D space, at least if our goal is to determine the most important mechanisms driving CFU dynamics.

We are also working on automating the second step for our model calibration, namely to sift out granulomas that properly form. Dissecting spatial structures that match our prototypical granuloma architecture will likely rely on more sophisticated techniques for volume matching or image comparisons. The goal will be to design and implement metrics with high specificity and sensitivity, in order to automatically screen among thousands of granulomas evolving over time.

Finally, when switching from a 2D to a 3D ABM environment, a more realistic representation of the lung vascularization (e.g., increased vascular source density) will enhance the model without significantly affecting the main outcomes. The downside could be the increased computational costs due to more agents entering the grid. The latter challenge could be overcome by introducing stricter thresholds for recruitment (without changing the recruitment probabilities). More experimental data will be needed to balance higher lung vascularization and cell densities within TB granulomas.

## Supplementary Material

Supplementary file 1

## Figures and Tables

**Table 1.** Implementation and high performance computing specifications.

	CPU h per 100 Days	Number of Micro-Compartments	Number of Agents [min-max]
**2D**	0.1–1.4 h	1 × 10^4^	[170, 9395]
**3D**	40–200 h	1 × 10^6^	[5 × 10^3^,1 × 10^6^]

**Table 2.** PRCC results for both 2D and 3D resolutions. We show the most important and significant (*p* <1 × 10^−3^) PRCC values at days 7,14, 28, 50, 60, 90 and 100 post infection. Although any PRCC between −0.15 and 0.15 can be considered not important, we show some of them for comparison purposes (**grey cells** highlight the 2D-3D overlap).

DAY 7	2D	GrowthRateIntMtb 0.9317	thresholdApoptosisT NF 0.3477	nrIntMtbBurstCInf 0.2505	Tcyt_maxRecProb 0.0561	probKillExtMt bRest −0.1016	kApoptosis −0.1423				
	3D	GGrowthRateIntMtb 0.9598	thresholdApoptosisT NF 0.2343	sourceDensity 0.1328	nrIntMtbBurstCInf 0.0412	estIntPartition TNF −0.0666	kApoptosis −0.0914	probKillExtMtbRest −0.1452			
DAY 14	2D	GrowthRateIntMtb 0.9382	thresholdApoptosisT NF 0.5081	nrIntMtbBurstCInf 0.3287	GrowthRateExtMtb 0.1506	estIntPartition TNF −0.1400	kApoptosis −0.2461				
	3D	GrowthRateIntMtb 0.9686	thresholdApoptosisT NF 0.4054	sourceDensity 0.1709	kDeg 0.0621	estIntPartition TNF −0.1138	kApoptosis −0.1787	probKillExtMtbRest −0.1906			
DAY 28	2D	GrowthRateIntMtb 0.9284	thresholdApoptosisT NF 0.5517	GrowthRateExtMt b 0.3578	nrIntMtbBurstCInf 0.3058	estIntPartition TNF −0.1559	kApoptosis −0.3047				
	3D	GrowthRateIntMtb 0.9655	thresholdApoptosisT NF 0.5348	GrowthRateExtMt b 0.2848	nrIntMtbBurstCInf 0.1570	estIntPartition TNF −0.1549	probKillExtMtb Rest −0.1871	kApoptosis −0.2982			
DAY 50	2D	GrowthRateIntMtb 0.7689	kDeg 0.3378	nrIntMtbBurstCInf 0.2926	maxRecProb 0.2819	Treg_maxRecP rob 0.2194	thresholdApopt osisTNF 0.2140	CC_kDeg −0.1687	probMoveT oMac −0.2763	sourceDensit y −0.2805	Tgam_maxRecP rob −0.3241
	3D	GrowthRateIntMtb 0.9163	kDeg 0.4921	Treg_maxRecProb 0.4660	thresholdApoptosisTNF 0.2145	Tgam_maxRec Prob −0.2028	dTNF −0.2217	factorDeactIL10 −0.2391			
DAY 60	2D	GrowthRateIntMtb 0.7442	kDeg 0.4008	maxRecProb 0.2992	nrIntMtbBurstCInf 0.2744	Treg_maxRecP rob 0.2400	thresholdApopt osisTNF 0.2011	CC_kDeg −0.1960	sourceDensi ty −0.2486	probMoveTo Mac −0.3073	Tgam_maxRecP rob −0.3241
	3D	GrowthRateIntMtb 0.8989	kDeg 0.4951	Treg_maxRecProb 0.4618	estConsRateTNF 0.2154	kNFkB −0.2019	dTNF −0.2108	factorDeact IL10 −0.2313		
DAY 90	2D	GrowthRateIntMtb 0.7010	kDeg 0.4419	maxRecProb 0.3058	Treg_maxRecProb 0.2379	nrIntMtbBurst Clnf 0.2297	estConsRateTN F 0.1927	CC_kDeg −0.2011	sourceDensi ty −0.2327	probMoveTo Mac −0.3132	TgammaxRecP rob −0.3342
	3D	GrowthRateIntMtb 0.8685	kDeg 0.5024	Treg_maxRecProb 0.4615	estConsRateTNF 0.2191	kNFkB −0.2028	factorDeactILl0 −0.2185	Ikdeg −0.2252		
DAY 100	2D	GrowthRateIntMtb 0.6931	kDeg 0.4471	maxRecProb 0.3019	Treg_maxRecProb 0.2374	nrIntMtbBurst Clnf 0.2210	estConsRateTN F 0.1914	CC_kDeg −0.1955	sourceDensi ty −0.2329	probMoveTo Mac −0.3081	TgammaxRecP rob −0.3306
	3D	GrowthRateIntMtb 0.8685	kDeg 0.5024	Treg_maxRecProb 0.4615	estConsRateTNF 0.2191	kNFkB −0.2028	factorDeactIL10 −0.2185	Ikdeg −0.2252		

## Box 1. Comparison of 2D, 3D, and merged versions of the code.

This pseudocode is about retrieving cell density information to use for diffusion of TNF, IL10, and the 3 chemokines. We use the C++ concept of an iterator to define grid iterators that have the same syntax for 2D and 3D. Since the syntax is the same, model code that iterates over the grid, or portions of it, is the same regardless of dimension.

**Table:**

2D specific:	3D specific:
{	{
GrGrid& g = _grid.getGrid();	GrGrid& g = _grid.getGrid();
Pos p;	Pos p;
Pos dim = g.getRange();	Pos dim = g.getRange();
for (p.x = 0; p.x<dim.x; p.x++)	for (p.x = 0; p.x<dim.x; p.x++)
{	{
for (p.y = 0; p.y<dim.y; p.y++)	for (p.y = 0; p.y<dim.y; p.y++)
{	{
g.nCells(p) = g.getCellDensity(p);	for (p.z = 0; p.z<dim.z; p.z++)
}}}	{
	g.nCells(p) = g.getCellDensity(p);
	}}}}

**Dimension independent:**

Get cell density information to use for diffusion

{

GrGrid& g = _grid.getGrid();

constPos& dim = g.getRange();

GridSpec::EntireGridIteratoregi = GridSpec::egiBegin(dim);

GridSpec::EntireGridIteratoregiEnd = GridSpec::egiEnd(dim);

for(; egi ! = egiEnd; ++egi)

{

constPos& p = *egi;

g.nCells(p) = g.getCellDensity(p);

}}
