# Supplementary material for: The Role of Dimensionality in Understanding Granuloma Formation
Source: Computation (Basel). Author manuscript; Available in PMC 2019 Jun 30. (PMC6599587; doi:10.3390/computation6040058)
Supplement: Supplementary file 1 [file NIHMS998495-supplement-Supplementary_file_1.pdf]

Supplementary Materials

The Role of Dimensionality in Understanding Granuloma Formation

Simeone Marino [1,2], Caitlin Hult [1,3], Paul Wolberg [1,3], Jennifer J. Linderman [3] and Denise E. Kirschner [1,4,*]

[1] Department of Microbiology and Immunology, University of Michigan Medical School, Ann Arbor, MI 48109, USA; simeonem@umich.edu (S.M.); cshult@umich.edu (C.H.); pwolberg@umich.edu (P.W.)

[2] Statistics Online Computational Resource (SOCR), Department of Health Behavior and Biological Sciences, University of Michigan, Ann Arbor, MI 48109, USA

[3] Department of Chemical Engineering, University of Michigan, Ann Arbor, MI 48109, USA; linderma@umich.edu

[4] Department of Computational Medicine and Bioinformatics, University of Michigan, Ann Arbor, MI 48109, USA

* Correspondence: kirschne@umich.edu

Table S1. List of all the parameters varied in the pilot uncertainty study, with the respective description and LHS range.

**Parameter Name**	**Range**	**Units**	**Description**
Mtb/@growthExtMtbBound	[180,240]	#bacteria	Upper bound of number of extMtb used in growth function
Mtb.Numbers/@growthRateIntMtb	[1.001, 1.006]	fraction of mtb/agent time step	Fractional growth rate of intracellular bacteria
Mtb.Numbers/@growthRateExtMtb	[1.001, 1.003]	fraction of mtb/agent time step	Fractional growth rate of extracellular bacteria
Mtb.Numbers/@deathRateExtMtbCaseated	[1,2]	1/agent time step	Upper bound on the number of extMtb used in growth function
Core/@estBoundFactorTNF	[0.4,0.5]	fraction of TNFR1 receptors that are TNF bound	Adjustment for coarse grained internalized fraction estimate
Core/@estBoundFactorIL10	[0.4,0.6]	?fraction of IL10R receptors that are IL10 bound	Adjustment for coarse grained internalized fraction estimate
Core/@estConsRateTNF	[5e-4,9e-4]	1/seconds	Scaling factor for coarse grained TNF dynamics
Core/@estConsRateIL10	[2e-4,6e-4]	1/seconds	Scaling factor for coarse grained IL10 dynamics
Core/@estIntPartitionTNF	[9,13]	?fraction of TNFR1 receptors that are internalized	Scaling factor for coarse grained internalization of boundTNFR1
Core/@nrKillingsCaseation	[7,13]	#cells	Number of killings for a compartment to become caseated
Core/@caseationHealingTime	[1700,2600]	#agent time steps	Time it takes for a caseated compartment to heal
Core/@sourceDensity	[0.002,0.05]	fraction of total grid compartments	Density of vascular sources on the grid
Core/@diffusivityTNF	[4e-08,6e-08]	cm^2/second	TNF diffusivity
Core/@diffusivityChemokines	[4e-08,6e-08]	cm^2/second	Chemokine diffusivity
Core/@diffusivityIL10	[4e-08,6e-08]	cm^2/second	IL10 diffusivity
Core/@ChemokinekDeg	[0.0005,0.005]	1/seconds	Chemokine degradation rate constant
Core/@kDeg	[0.0005,0.005]	1/seconds	TNF degradation rate constant
Core/@Ikdeg	[0.0003,0.003]	1/seconds	Degredation rate constant for ODE IL10
Core/@IC50ChemokineIL10	[1,10]	#molecules/milli-liter	IC50 of IL10 inhibition of chemokine secretion - All chemokines are the same
Core/@thresholdApoptosisTNF	[1000,5000]	#internalized TNF-bound TNFR1	TNF threshold for TNF-induced apoptosis
Core/@kApoptosis	[1e-07,2e-6]	1/seconds	Rate of TNF-induced apoptosis
Core/@saturationApoptosisTNF	[5000,9000]	#molecules	Signal saturation of number of int-bound TNFR1 Molecules

Core/@minChemotaxis	[1,50]	#molecules	Chemotaxissensivity range (lower bound)
Core/@maxChemotaxis	[100,1000]	#molecules	Chemotaxissensivity range (upper bound)
Core/@MaxIL10Inhibition	[0.05,0.3]	log10(nanograms/milli-liter)	Coarse grained TNF/IL10 dose dependence parameter beta
Core.Mac/@initDensity	[0.005,0.03]	fraction of total grid compartments	Initial density of resting macrophages on the grid
Core.Mac/@movementRest	[1,10]	#agent time steps	Time required for a resting macrophage to move one micro-compartment
Core.Mac/@movementAct	[10,50]	#agent time steps	Time required for an active macrophage to move one micro-compartment
Core.Mac/@movementInf	[100,200]	#agent time steps	Time required for an infected macrophage to move one micro-compartment
Core.Mac/@dTNF	[1.3,1.7]	#molecules/second	Secretion rate of TNF by Mac
Core.Mac/@dCCL2	[4,8]	#molecules/second	Secretion rate of CCL2 by Mac
Core.Mac/@dCCL5	[4,8]	#molecules/second	Secretion rate of CCL5 by Mac
Core.Mac/@dCXCL9	[10,14]	#molecules/second	Secretion rate of CXCL9 by Mac
Core.Mac/@dIL10Act	[0.2,0.4]	#molecules/second	Secretion rate of IL10 by Mac Act
Core.Mac/@dIL10Inf	[0.01,0.03]	#molecules/second	Secretion rate of IL10 by Mac Inf
Core.Mac/@halfSatIL10	[170,210]	#molecules	Half Sat for TNF induction of IL10 in activated Macs
Core.Mac/@thresholdNFkBTNF	[75,115]	#molecules	TNF threshold for NFkB activation
Core.Mac/@kNFkB	[0.7e-5,1e-5]	1/seconds	Rate of NFkB activation
Core.Mac/@probKillExtMtbRest	[0.05, 0.3]	probability	Probability of a resting macrophage to kill some extracellular bacteria
Core.Mac/@fKillExtMtbRest	[0.3, 0.5]	fractional increase of a STAT1 or NFkB stimualted resting mac's probability to kill an extmt	Fractional increase of a resting macrophage to kill some extracellular bacteria when STAT1 || NFkB
Core.Mac/@nrExtMtbNFkB	[150, 250]	#bacteria	Number of extracellular bacteria for NFkB activation in an infected macrophage
Core.Mac/@nrIntMtbCInf	[8,12]	#bacteria	Number of intracellular bacteria for an infected macrophage to become chronically infected
Core.Mac/@nrIntMtbBurstCInf	[13, 20]	#bacteria	Number of intracellular bacteria for an chronically infected macrophage to burst

Core.Mac/@nrExtMtbUptakeAct	[3, 7]	#bacteria	Number of extracellular bacteria an active macrophage can uptake and subsequently kill
Core.Mac/@stat1ActivationTime	[400, 460]	#agent time steps	Time interval a Macrophage is STAT1 Activated
Core.Mac/@nfkbActivationTime	[13, 17]	#agent time steps	Time a Macrophage is NFkB Activated
Core.Mac/@stat3ActivationTime	[75, 125]	#agent time steps	Time a Macrophage is STAT3 Activated
Core.Mac/@thresholdSTAT3IL10	[5, 15]	#molecules	Threshold of bound IL10 to IL10R1 for STAT3 signaling - Non-Molecular
Core.Mac/@kSTAT3IL10	[5e-4, 1.5e-3]	1/(#molecules * agent time step)	Rate constant of bound IL10 to IL10R1 for STAT3 signaling - Non-Molecular
Core.Mac/@probHealCaseation	[0.005, 0.05]	probability	Rate constant for wound healing - Non-Molecular
Core.Tcell/@maxAge	[400, 460]	#agent time steps	Maximal T cell age
Core.Tcell/@exhaustedMaxAge	[400, 460]	#agent time steps	Maximal T cell age of an exhausted T cell
Core.Tcell/@probMoveToMac	[0.01, 0.2]	probability	Probability of a T cell moving onto a compartment already containing a macrophage
Core.Tcell/@probMoveToTcell	[0.01, 0.2]	probability	Probability of a T cell moving onto a compartment already containing another T cell
Core.Tcell/@maxDivisions	[3, 5]	#Tcell divisions	Maximum number of divisions: max number of times a T cell can create a daughter cell
Core.Tcell.Tgam/@dTNF	[0.1, 0.2]	#molecules/second	Secretion rate of TNF by Tgam
Core.Tcell.Tgam/@maxTimeReg	[30, 40]	#agent time steps	Time span during which a Tgam cell remains down-regulated
Core.Tcell.Tgam/@probApoptosisFasFasL	[0.01, 0.03]	Probability	Probability of Fas/FasL induced apoptosis by a Tgam cell
Core.Tcell.Tgam/@probTNFProducer	[0.04, 0.1]	Probability	Probability that this Tgam is producing TNF
Core.Tcell.Tgam/@probIFNProducer	[0.3, 0.4]	Probability	Probability that this Tgam is producing TNF
Core.Tcell.Tgam/@probIFNMooreExtend	[0.20, 0.3]	Probability	Probability Mac will be IFN/STAT1 activated in the extended Moore
Core.Tcell.Tcyt/@dTNF	[0.01, 0.02]	#molecules/second	Secretion rate of TNF by Tcyt
Core.Tcell.Tcyt/@maxTimeReg	[30, 40]	#agent time steps	Time span during which a Tcyt cell remains down-regulated
Core.Tcell.Tcyt/@probKillMac	[0.005, 0.015]	Probability	Probability of a Tcyt cell killing a (chronically) infected macrophage
Core.Tcell.Tcyt/@probKillMacCleanly	[0.6, 0.9]	Probability	Probability of a Tcyt cell killing a chronically infected macrophage cleanly

Core.Tcell.Tcyt/@probTNFProducer	[0.05, 0.09]	Probability	Probability that this Tcyt is producing TNF
Core.Tcell.Treg/@dIL10	[0.7, 0.8]	#molecules/second	Secretion rate of IL10 by Treg
Core.Tcell.Treg/@probTregDeactivate	[0.01, 0.02]	probability	Probability of successful downregulation by a Treg
Core.Tcell.Treg/@factorDeactIL10	[1,3]	unitless	Factor when a Treg is making IL-10 to scale probTregDeactivate
Recruit.Prob/@timeMaxRecDelay	[500,900]	#agent time steps	Time after which maximum T cell recruitment is enabled
Recruit.Prob.Mac/@maxRecProb	[0.1, 0.4]	probability	Max probability of recruiting a macrophage - Scales recruitment function
Recruit.Prob.Mac/@thresholdRecChemokine	[0.015, 1.5]	#molecules	Threshold of Mac Recruitment for Chemokines
Recruit.Prob.Mac/@thresholdRecTNF	[0.005, 0.5]	#molecules	Threshold of Mac Recruitment for TNF
Recruit.Prob.Mac/@recruitmentHalfSatTNF	[0.055, 5.5]	#molecules	Half-saturation paramter for Mac TNF-dependent recruitment
Recruit.Prob.Mac/@recruitmentHalfSatChemokine	[1, 10]	#molecules	Half-saturation paramter for Mac chemokine-dependent recruitment
Recruit.Prob.Tcell.Tgam/@maxRecProb	[0.05, 0.15]	probability	Max probability of recruiting a Tgam - Scales recruitment function
Recruit.Prob.Tcell.Tgam/@thresholdRecChemokine	[0.0151, 1.5]	#molecules	Threshold of Tgam Recruitment for Chemokines
Recruit.Prob.Tcell.Tgam/@thresholdRecTNF	[0.01, 1.0]	#molecules	Threshold of Tgam Recruitment for TNF
Recruit.Prob.Tcell.Tgam/@recruitmentHalfSatTNF	[0.04, 4.0]	#molecules	Half-saturation paramter for Tcell TNF-dependent recruitment
Recruit.Prob.Tcell.Tgam/@recruitmentHalfSatChemokine	[0.15, 15]	#molecules	Half-saturation paramter for Tcell chemokine-dependent recruitment
Recruit.Prob.Tcell.Tcyt/@maxRecProb	[0.03, 0.13]	Probability	Max probability of recruiting a Tcyt - Scales recruitment function
Recruit.Prob.Tcell.Tcyt/@thresholdRecChemokine	[0.03775, 3]	#molecules	Threshold of TCYT Recruitment for Chemokines
Recruit.Prob.Tcell.Tcyt/@thresholdRecTNF	[0.01, 1.0]	#molecules	Threshold of TCYT Recruitment for TNF
Recruit.Prob.Tcell.Tcyt/@recruitmentHalfSatTNF	[0.04, 4.0]	#molecules	Half-saturation paramter for Tcell TNF-dependent recruitment
Recruit.Prob.Tcell.Tcyt/@recruitmentHalfSatChemokine	[0.15, 15]	#molecules	Half-saturation paramter for Tcell chemokine-dependent recruitment

Recruit.Prob.Tcell.Treg/@maxRecProb	[0.0075, 0.075]	probability	Max probability of recruiting a Treg - Scales recruitment function
Recruit.Prob.Tcell.Treg/@thresholdRecChemokine	[0.00755, 0.7]	#molecules	Threshold of TREG Recruitment for Chemokines
Recruit.Prob.Tcell.Treg/@thresholdRecTNF	[0.01, 1.0]	#molecules	Threshold of TREG Recruitment for TNF
Recruit.Prob.Tcell.Treg/@recruitmentHalfSatTNF	[0.04, 4.0]	#molecules	Half-saturation paramter for Tcell TNF-dependent recruitment
Recruit.Prob.Tcell.Treg/@recruitmentHalfSatChemokine	[0.15, 15]	#molecules	Half-saturation paramter for Tcell chemokine-dependent recruitment

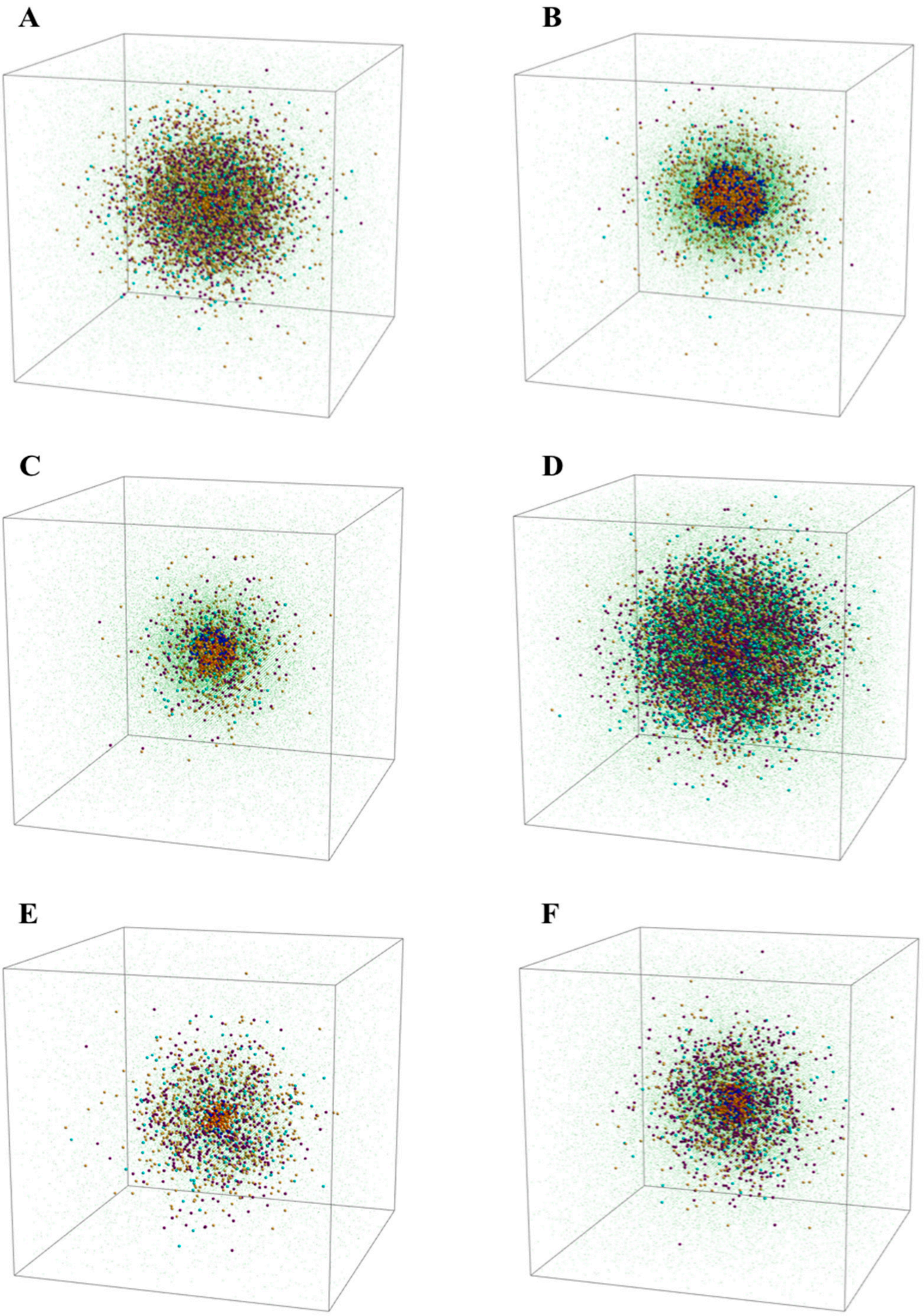

Figure S1. Examples of other *in silico* granulomas, visualized in 3D. Here we visualize *in silico* granulomas corresponding to eight of the 22 parameter files determined through the CFU calibration described in the Methods and Results section. These images illustrate some of the variation possible among the parameter files. Granulomas correspond to day 32 post infection. The list of the 90 parameters corresponding to each granuloma are shown in Table S2 in the Supplementary Material.

Table S2. list of the 90 parameters values corresponding to each granuloma shown in Figure S1. The experiment numbers shown refer to the correspondent parameter sample (out of 1,500 samples) generated by the Latin hypercube sampling scheme.

Parameter Name	Exp107	Exp1217	Exp1331	Exp588	Exp1099	Exp1460
Mtb/@growthExtMtbBound	212.7036	190.2303	226.7785	212.3112	216.5995	208.039
Mtb.Numbers/@growthRateIntMtb	1.001955	1.00332	1.002095	1.00286	1.001791	1.001946
Mtb.Numbers/@growthRateExtMtb	1.002698	1.001362	1.002077	1.002509	1.002293	1.002236
Mtb.Numbers/@deathRateExtMtbCaseated	1.73065	1.007751	1.839851	1.423186	1.949764	1.69439
Core/@estBoundFactorTNF	0.431709	0.463145	0.463788	0.42911	0.414548	0.44913
Core/@estBoundFactorIL10	0.484486	0.575668	0.510042	0.493809	0.438566	0.464123
Core/@estConsRateTNF	0.000844	0.000578	0.000868	0.000802	0.00073	0.000888
Core/@estConsRateIL10	0.000509	0.000342	0.000254	0.0004	0.000465	0.000522
Core/@estIntPartitionTNF	11.66407	12.57022	9.003913	10.61256	12.93329	9.716339
Core/@nrKillingsCaseation	8	9	13	8	7	7
Core/@caseationHealingTime	2250	2598	1834	2499	2295	2457
Core/@sourceDensity	0.031379	0.045881	0.047061	0.047537	0.006303	0.01274
Core/@diffusivityTNF	5.7E-08	4.11E-08	5.76E-08	4.28E-08	4.95E-08	4.7E-08
Core/@diffusivityChemokines	4.04E-08	5.66E-08	5.39E-08	5.08E-08	4.7E-08	5.21E-08
Core/@diffusivityIL10	4.09E-08	5.6E-08	4.82E-08	5.06E-08	4.78E-08	5.52E-08
Core/@ChemokinekDeg	0.001173	0.003544	0.003088	0.001213	0.000677	0.001754
Core/@kDeg	0.004089	0.004835	0.003819	0.00222	0.004528	0.003773
Core/@Ikdeg	0.001559	0.002579	0.00089	0.002063	0.002785	0.000909
Core/@IC50ChemokineIL10	5.986183	7.494756	4.081917	4.651744	9.853524	7.519626
Core/@thresholdApoptosisTNF	3768.642	3834.609	3371.569	2061.799	4096.084	1422.949
Core/@kApoptosis	1.64E-06	1.73E-06	1.55E-06	2.86E-07	1.78E-06	2.02E-07
Core/@saturationApoptosisTNF	8296.478	5055.741	8394.854	6674.25	6964.417	8877.03
Core/@minChemotaxis	26.10367	13.74456	33.17806	17.66427	12.74171	12.47196
Core/@maxChemotaxis	716.5819	714.0672	453.7457	659.3666	213.8385	728.2961
Core/@MaxIL10Inhibition	0.156721	0.056617	0.28328	0.27405	0.210947	0.121587
Core.Mac/@initDensity	0.013239	0.007467	0.014246	0.027988	0.009458	0.01706
Core.Mac/@movementRest	3	6	4	3	9	3
Core.Mac/@movementAct	49	42	38	27	11	33
Core.Mac/@movementInf	123	113	123	144	175	153
Core.Mac/@dTNF	1.373567	1.52956	1.60419	1.695448	1.467669	1.558262
Core.Mac/@dCCL2	4.909274	5.895709	4.794036	6.306414	5.774316	5.632097
Core.Mac/@dCCL5	4.47138	4.943601	7.567779	7.016468	5.62262	6.173624

Core.Mac/@dCXCL9	12.73673	10.04317	12.44619	10.43985	11.04102	13.88383
Core.Mac/@dIL10Act	0.344593	0.226707	0.387289	0.288056	0.369029	0.207422
Core.Mac/@dIL10Inf	0.023796	0.028697	0.019229	0.011304	0.027456	0.024041
Core.Mac/@halfSatIL10	208.5436	202.9831	208.1734	193.1971	205.0046	184.1318
Core.Mac/@thresholdNFkBTNF	108.1988	76.39743	80.25483	83.68737	87.04637	84.98787
Core.Mac/@kNFkB	9.92E-06	9.85E-06	7.33E-06	8.96E-06	8.17E-06	7.89E-06
Core.Mac/@probKillExtMtbRest	0.236164	0.243177	0.213914	0.125766	0.225209	0.156869
Core.Mac/@fKillExtMtbRest	0.33891	0.318811	0.336385	0.436383	0.316346	0.485938
Core.Mac/@nrExtMtbNFkB	203.5147	232.828	213.6564	187.7995	151.7827	242.0825
Core.Mac/@nrIntMtbCInf	12	12	9	8	11	11
Core.Mac/@nrIntMtbBurstCInf	18	19	13	20	19	14
Core.Mac/@nrExtMtbUptakeAct	4	3	5	3	4	4
Core.Mac/@stat1ActivationTime	438.74	455.9144	449.9	459.7025	438.8812	452.404
Core.Mac/@nfkbActivationTime	14.16019	16.42173	13.23257	14.23056	16.21124	14.84746
Core.Mac/@stat3ActivationTime	114.3614	88.32152	104.3877	124.7755	89.6549	105.7309
Core.Mac/@thresholdSTAT3IL10	9.737525	13.11575	13.86993	6.789302	12.12305	11.70058
Core.Mac/@kSTAT3IL10	0.00139	0.001232	0.001019	0.000713	0.001193	0.000849
Core.Mac/@probHealCaseation	0.014079	0.024336	0.049197	0.027745	0.028963	0.036337
Core.Tcell/@maxAge	411	434	424	432	411	402
Core.Tcell/@exhaustedMaxAge	443	424	433	413	409	413
Core.Tcell/@probMoveToMac	0.074868	0.042021	0.027888	0.077404	0.12059	0.088926
Core.Tcell/@probMoveToTcell	0.025554	0.012214	0.032102	0.163135	0.083153	0.010266
Core.Tcell/@maxDivisions	3	3	3	3	5	4
Core.Tcell.Tgam/@dTNF	0.197828	0.173356	0.127363	0.187541	0.157129	0.126285
Core.Tcell.Tgam/@maxTimeReg	40	37	37	39	39	35
Core.Tcell.Tgam/@probApoptosisFasFasL	0.01646	0.018783	0.02629	0.012317	0.018548	0.018535
Core.Tcell.Tgam/@probTNFProducer	0.083465	0.050492	0.093335	0.056354	0.057832	0.045978
Core.Tcell.Tgam/@probIFNProducer	0.37901	0.338223	0.319791	0.371618	0.38481	0.301018
Core.Tcell.Tgam/@probIFNMooreExtend	0.228199	0.243467	0.206911	0.279134	0.268253	0.26071
Core.Tcell.Tcyt/@dTNF	0.014435	0.019764	0.01376	0.016656	0.016756	0.019005
Core.Tcell.Tcyt/@maxTimeReg	39	38	39	32	34	37
Core.Tcell.Tcyt/@probKillMac	0.013302	0.012353	0.007546	0.008492	0.01136	0.012423
Core.Tcell.Tcyt/@probKillMacCleanly	0.886567	0.623039	0.890478	0.755467	0.635762	0.640955
Core.Tcell.Tcyt/@probTNFProducer	0.086863	0.066919	0.084475	0.069241	0.066652	0.070682
Core.Tcell.Treg/@dIL10	0.773153	0.798214	0.705819	0.737648	0.746762	0.756761
Core.Tcell.Treg/@probTregDeactivate	0.014375	0.011237	0.016095	0.014172	0.01214	0.017359

Core.Tcell.Treg/@factorDeactIL10	2.899996	2.6052	1.660427	1.292824	1.398151	2.641041
Recruit.Prob/@timeMaxRecDelay	740	554	851	617	645	562
Recruit.Prob.Mac/@maxRecProb	0.311086	0.357383	0.277989	0.287472	0.140233	0.365722
Recruit.Prob.Mac/@thresholdRecChemokine	0.844973	1.299753	0.288299	1.063154	0.567517	0.409053
Recruit.Prob.Mac/@thresholdRecTNF	0.32985	0.328308	0.346965	0.196217	0.01885	0.476191
Recruit.Prob.Mac/@recruitmentHalfSatTNF	2.013039	2.80207	2.313902	1.689667	4.445079	4.003354
Recruit.Prob.Mac/@recruitmentHalfSatChemokine	9.336256	3.056593	6.790684	9.098188	6.538349	5.050239
Recruit.Prob.Tcell.Tgam/@maxRecProb	0.113713	0.054449	0.115355	0.0585	0.070061	0.079866
Recruit.Prob.Tcell.Tgam/@thresholdRecChemokine	0.104409	0.179187	1.100593	0.492282	0.710556	0.513107
Recruit.Prob.Tcell.Tgam/@thresholdRecTNF	0.833406	0.856212	0.9306	0.234757	0.9124	0.288354
Recruit.Prob.Tcell.Tgam/@recruitmentHalfSatTNF	0.040101	3.369557	3.622599	1.723027	0.953238	1.924597
Recruit.Prob.Tcell.Tgam/@recruitmentHalfSatChemokine	8.499244	11.20854	13.40532	10.91517	7.0765	9.254699
Recruit.Prob.Tcell.Tcyt/@maxRecProb	0.116013	0.040857	0.06603	0.099326	0.098067	0.088366
Recruit.Prob.Tcell.Tcyt/@thresholdRecChemokine	1.995664	1.663344	1.241825	1.831602	1.829797	1.621107
Recruit.Prob.Tcell.Tcyt/@thresholdRecTNF	0.742341	0.759802	0.294099	0.186856	0.209493	0.281977
Recruit.Prob.Tcell.Tcyt/@recruitmentHalfSatTNF	1.182729	1.921854	2.932541	3.602318	3.510149	1.085081
Recruit.Prob.Tcell.Tcyt/@recruitmentHalfSatChemokine	4.760701	12.63024	8.863514	4.184256	13.54121	1.145628
Recruit.Prob.Tcell.Treg/@maxRecProb	0.061887	0.032558	0.040755	0.044468	0.030325	0.050833
Recruit.Prob.Tcell.Treg/@thresholdRecChemokine	0.375637	0.407376	0.489457	0.500638	0.101731	0.539927
Recruit.Prob.Tcell.Treg/@thresholdRecTNF	0.054114	0.102815	0.939653	0.813379	0.04119	0.931099
Recruit.Prob.Tcell.Treg/@recruitmentHalfSatTNF	3.205315	3.839902	3.859628	3.317244	1.653953	1.676516
Recruit.Prob.Tcell.Treg/@recruitmentHalfSatChemokine	14.48033	13.57721	10.82076	2.082219	6.314493	4.472219
